# A surface reconstruction route for increasingly improved photocatalytic H_2_O_2_ production using Sr_2_Bi_3_Ta_2_O_11_Cl†

**DOI:** 10.1039/d4sc04866k

**Published:** 2024-09-18

**Authors:** Maqsuma Banoo, Arjun Kumar Sah, Raj Sekhar Roy, Komalpreet Kaur, Bramhaiah Kommula, Dirtha Sanyal, Ujjal K. Gautam

**Affiliations:** a Department of Chemical Sciences, Indian Institute of Science Education and Research (IISER)-Mohali Sector 81, Mohali, S.A.S. Nagar Punjab 140306 India ujjalgautam@iisermohali.ac.in ujjalgautam@gmail.com; b Variable Energy Cyclotron Centre 1/AF Bidhannagar Kolkata 700064 India; c Homi Bhabha National Institute Anushakti Nagar Mumbai 400094 India

## Abstract

Photocatalytic hydrogen peroxide (H_2_O_2_) generation is attractive for the chemical industry and energy production. However, photocatalysts generally deteriorate significantly during use to limit their application. Here we present highly active Sr_2_Bi_3_Ta_2_O_11_Cl single-crystal nanoplates for conversion of O_2_ to H_2_O_2_ using ambient air with a production rate of ∼3 mmol h^−1^ g^−1^ (maximum 17.5% photon conversion). Importantly, Sr_2_Bi_3_Ta_2_O_11_Cl is not only stable during 30 days of H_2_O_2_ production but also gets consistently activated to increase the H_2_O_2_ yield by >244%, unlike any other catalyst for H_2_O_2_ production. Multi-pronged characterization confirms that the synergistic increase in activity originates from *in situ* surface reconstruction by oxygen-deficient vacancy associate formation that improves (i) surface oxygen adsorption, (ii) sunlight harvesting, and (iii) charge-transfer from the low-valent metal atoms surrounding oxygen vacancies to reactants. The study establishes the prospects of rational defect engineering for realizing non-degrading photocatalysts for realistic H_2_O_2_ production.

## Abstract 2

Sr_2_Bi_3_Ta_2_O_11_Cl as an excellent photocatalyst for H_2_O_2_ production (∼3 mmol h^−1^ g^−1^) that further improves as time passes due to surface vacancy formation, demonstrating the role of rational defect engineering in developing sustainable photocatalysts.

## Graphical abstract

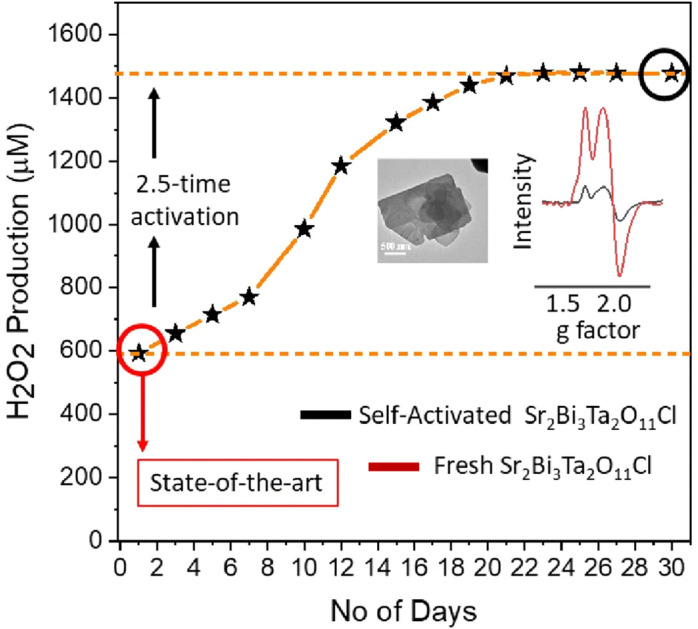

## 1 Introduction

Improved production of H_2_O_2_ is highly desirable due to its extensive utilization as a clean, low-cost, environment-friendly oxidant and high-energy density fuel.^1–3^ However, conventional industrial approaches such as the anthraquinone oxidation route are not environmentally benign because of the utilization of high-energy multistep reactions and safety concerns while handling high-pressure hydrogen.^4,5^ In this context, photocatalytic H_2_O_2_ production using light-harvesting semiconductors is highly promising and has attracted widespread research attention recently, leading to the development of several facile catalytic materials, including metal oxides, sulfides, nitrides, metal-free materials, *etc.*^6–11^ Even though progress in developing highly active materials is rapid, a serious drawback with photocatalysts, in general, is the degradation of their catalytic activities with continued use, while industries spend billions per year just for their replacement.

Photocatalyst deactivation usually originates from chemical deactivation, such as leaching or mechanical deactivation.^12,13^ Upon careful investigation, it is now becoming possible to develop activity restoration strategies for a handful of photocatalysts by using thermal or ultrasonic annealing, or by cocatalyst reloading.^14–16^ However, long-term stability studies and restoration of catalytic activity in photocatalysts used for H_2_O_2_ production are yet to be undertaken. In this scenario, the realization of a semiconductor photocatalyst with high catalytic efficiency and further self-activating properties instead of deactivation will be a meaningful contribution to a sustainable goal.

A careful analysis of the recent literature reveals that anion defect introduction is a preferred means to improve H_2_O_2_ production because molecular oxygen activation and dissociation are highly favourable at oxygen vacancies.^17–19^ Besides, defects can alter the catalyst's electronic structure also for harnessing more visible light and suppressing the recombination of excitons.^19,20^ However, the atomic-level healing of anion vacancies during oxygen reduction would macroscopically reoxidize the oxide surface and thus block molecular oxygen activation.^18^ Therefore, self-healing kinetics in the material should ideally be overcompensated for by ion leaching dynamics at the surface.

The Sillen–Aurivillius (SA) oxide phases of layered perovskites are interesting due to several advantages over conventional photocatalysts. The SA phase consists of a layer sequence of fluorite [Bi_2_O_2_], perovskite [A_*n*−1_B_*n*_O_3*n*+1_], fluorite [Bi_2_O_2_], and halide [X] layers (where A: Ca^2+^, Sr^2+^, Bi^3+^, *etc.*; B: Nb^5+^, Ta^5+^, Ti^4+^, *etc.*; X: halide; *n* = 1,2,3, *etc.*, the number of perovskite layers).^21–23^ Their advantages include high photostability and a narrow band gap (2.1–2.7 eV) unlike other mixed anion metal oxides, besides a high piezoresponse with many potential applications.^24–28^ The unique optical properties are attributed to the unusual occupation of a highly dispersive O 2p orbital near valence band maxima (V.B.M.) originating from a small Madelung potential at the oxygen sites in the fluorite-based [Bi_2_O_2_] layer and sizable interaction between O 2p and Bi 6s orbitals.^29,30^ The immense structural flexibility originating from the selection possibility of different stacking sequences of the Sillen and Aurivillius layers makes S. A. phases promising for many photocatalytic applications. Besides, the compositional versatility of the cations with different valences and the presence or absence of lone-pair electrons becomes more advantageous when *n* is of a higher order than its basic and well-explored *n* = 1 system. In contrast to Bi_4_MO_8_X (*n* = 1), which has almost similar *E*_CBM_ and *E*_VBM_ for different X and M, *n* = 2,3… *etc.* compounds have highly tailorable *E*_CBM_ and *E*_VBM_.^24^ Such a compound with *n* = 2, Sr_2_Bi_3_Ta_2_O_11_Cl has an electron mobility of 1.76 × 10^−7^ m^2^ V^−1^ s^−1^ and a more negative C.B.M. (−0.55 eV) than molecular oxygen reduction potential making it promising for photocatalytic H_2_O_2_ production.^31^

**Fig. 1:**
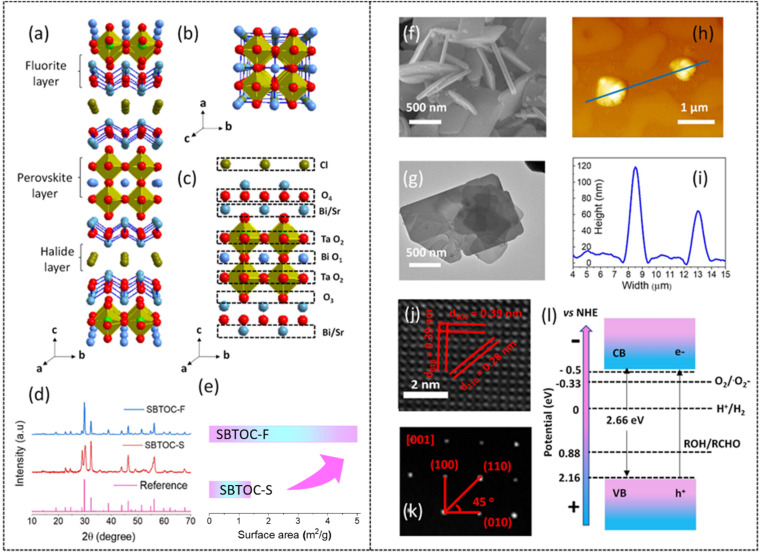
Crystal structure of Sr_2_Bi_3_Ta_2_O_11_Cl viewed from the (a) *bc* plane and (b) *ab* plane. Blue, light green, red, and dark green balls represent Bi, Ta, O, and Cl atoms, respectively. (c) The different types of lattice oxygen in Sr_2_Bi_3_Ta_2_O_11_Cl. (d) PXRD patterns and (e) surface area of SBTOC-F and SBTOC-S. (f and g) Scanning and transmission electron microscopy images of the nanoplates. (h and i) Atomic force microscopy image and the corresponding height profile, respectively. (j) HRTEM image and (k) single-crystalline SAED pattern acquired on 001 facets of a Sr_2_Bi_3_Ta_2_O_11_Cl nanoplate. (l) Schematic diagram of the experimentally determined energy levels for SBTOC-F.

Sr_2_Bi_3_Ta_2_O_11_Cl has a non-centrosymmetric (pseudo-tetragonal) crystal structure with a space group of *P*4/*mmm* (Fig. 1a–c). It consists of four different lattice oxygen sites. O_1_ and O_2_ are parts of the 12-coordinated Bi-alone site, distorting the polyhedron due to the presence of a stereochemically active Bi 6s^2^ lone pair and the octahedral off-centering of the adjacent Ta octahedra from the second-order Jahn–Teller effect. The O_3_ is distorted to a rather high extent at the interface between the [BiSrO_2_]^+^ layer and the perovskite block, while O_4_ is present toward the halide layer. This distortion-led Sr_2_Bi_3_Ta_2_O_11_Cl possesses inherent ferroelectric polarization and contributes to efficient electron and hole separation along the (001) and (100) directions, respectively.^31–33^ Because hole scavenging is rate-determining for H_2_O_2_ production (from O_2_) that can be greatly accelerated by using alcohols as hole-scavengers, enhancing those facets that the holes migrate can lead to enhanced H_2_O_2_ production. Recent studies have established that the holes preferentially migrate to the [001] facets of a SA nanoplate while the electrons migrate to the edges; therefore, an improved ratio of the [001]/[100] surface area can be deemed to improve H_2_O_2_ production.^34^ The traditional solid-state method of synthesizing SA phases has the limitations of uncontrolled facet-less growth and significantly low surface area (1–3 m^2^ g^−1^) observed at high-temperature sintering, while lower temperature leads to impurity phases. The flux method, on the other hand, can reduce their synthesis temperature due to an increase in the rate of ion diffusion to make the process sustainable and can also control the growth of specific facets.^35^ This possibility has not been explored for the *n* = 2 Sr_2_Bi_3_Ta_2_O_11_Cl phase yet. The other factor that can result in high H_2_O_2_ production is the abundance of adsorbed molecular O_2_ on the catalyst surface, which is usually less due to poor oxygen solubility in water. O_2_ molecules absorb on an oxide surface preferably at the oxygen vacancies due to an excess electron density that transfers to oxygen having a low reduction potential to stabilize it. We recently observed the facile formation of oxygen vacancies in the SA phases during photocatalysis originating from the Bi_2_O_2_ layers while the Aurivillius layers with strong M−O bonds maintain structural stability.^22^ Therefore, SA phases can potentially exhibit a high H_2_O_2_ photo-production rate that does not deteriorate or remain stable but rather increases further with time. Examples of such self-activating photocatalysts are rare.

Herein, we report highly active facet-controlled Sr_2_Bi_3_Ta_2_O_11_Cl nanoplates enclosed with the [100], [010], and [001] facets for efficient exciton separation by using a flux method for the first time. The single-crystal nanoplates have significantly high surface area and facet area ratios of [001]/[100] or [001]/[010] along which the excitons separate more than that achievable by the traditional approaches and photocatalytically produce H_2_O_2_ with remarkable efficiency even without an oxygen atmosphere. The amount of H_2_O_2_ produced is ∼2.9 mmol h^−1^ g^−1^, at par with state-of-the-art catalysts, and further improves by oxygen purging. More importantly, we established that due to surface reconstruction from the formation of oxygen vacancies and Bi leaching during continuous use, the catalyst exhibits self-activation behavior and can increase the H_2_O_2_ production by ∼244% after 30 days of sunlight exposure, unlike common photocatalysts that exhibit deactivation. The oxygen vacancies formed during the reuse play a decisive role in molecular oxygen activation along with boosting carrier separation and transfer.

## 2 Results and discussion

### 2.1 Catalyst characterization

The double layer perovskite Sillen–Aurivillius phase of Sr_2_Bi_3_Ta_2_O_11_Cl, where *n* = 2, was realized by using a eutectic mixture of NaCl and KCl (SBTOC-F, Scheme 1 in the ESI†) due to several advantages of the flux synthesis approach (ESI note 1†) and also by the traditional solid-state (SBTOC-S) method for comparison.^35,36^ Powder X-ray diffraction (PXRD, Fig. 1d and S1†) analysis confirms the purity of the samples.^27^ The sharp peaks of SBTOC-F and altered peak intensity patterns indicate the high crystallinity of the flux-synthesized sample and an interesting restricted growth orientation along (001) (ESI Note 2†). N_2_ adsorption–desorption isotherm analysis shows that the surface area of SBTOC-F (5.0 ± 0.3 m^2^ g^−1^) exhibits more than threefold enhancement over SBTOC-S (∼1.38 ± 0.1 m^2^ g^−1^Fig. 1e and S2a†). This was further affirmed by scanning electron microscopy (SEM, Fig. 1f), transmission electron microscopy (TEM, Fig. 1g), and atomic force microscopy (AFM, Fig. 1h and i) analyses which revealed that SBTOC-F consists of well-defined rectangular-shaped nanoplates having an average edge length of ∼1 μm and 60–100 nm of heights, whereas SBTOC-S consists of large irregularly shaped particles (Fig. S2b and c†). The high-resolution TEM (HRTEM) images acquired on the nanoplate (Fig. 1j) confirmed the high crystallinity of the nanoplates having lattice fringes (∼0.28 and ∼0.39 nm) corresponding to the (110) and (010) planes respectively and therefore, consideration of the uniform plate thickness indicated the dominantly exposed facet as {001}. The selected area electron diffraction (SAED, Fig. 1k) recorded on a single nanoplate along the [001] zone axis contained sharp diffraction spots indexable on its crystal structure, depicting the single crystalline nature of SBTOC-F and confirming the growth direction (Fig. S2d†). An HRTEM image and the SAED pattern of SBTOC-S are shown in Fig. S2e and f† to display the polycrystalline nature of the particles. The average band gaps for SBTOC-F and SBTOC-S were estimated at ∼2.66 eV and ∼2.7 eV, with the valence and conduction bands positioned at 2.16 and −0.50 eV *vs.* RHE respectively for SBTOC-F, indicating visible light sensitivity of the samples (Fig. 1l, S3a and b†).^31^

### 2.2 Photocatalytic hydrogen peroxide generation

**Fig. 2:**
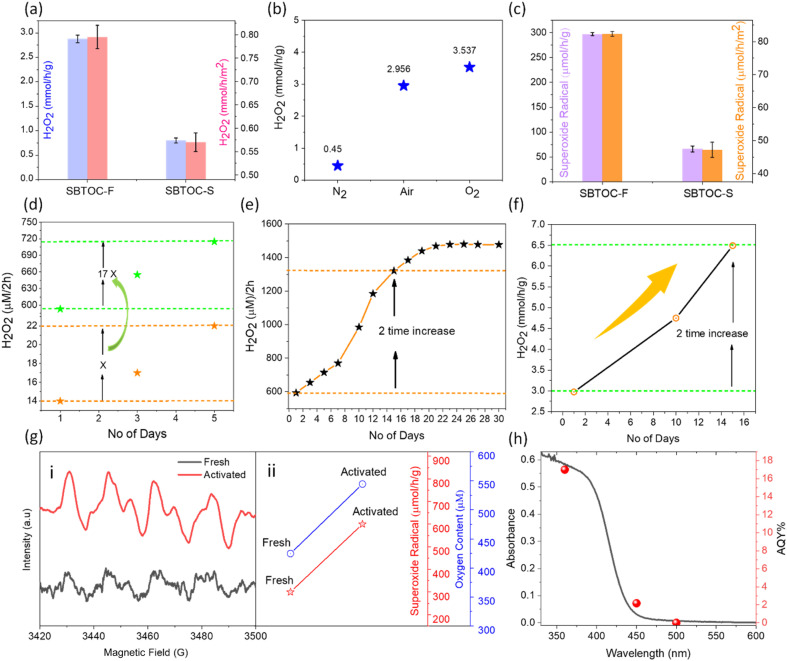
(a and b) The initial H_2_O_2_ production comparison among SBTOC-S and SBTOC-F in a 10% ethanol–water mixture. (b) H_2_O_2_ production by the SBTOC-F photocatalyst under different atmospheres in the 10% ethanol–water mixture. (c) Comparison of superoxide radical production efficiency by SBTOC-S and SBTOC-F. (d) Plot showing 17 times more self-activation in SBTOC-F during H_2_O_2_ production as compared to SBTOC-S in pure water. (e) Performance of SBTOC-F during continuous use for 30 days in pure water. (f) Performance of SBTOC-F during continuous use for 15 days in the 10% ethanol–water mixture. (g(i)) EPR spectra of DMPO-O_2_^˙−^ over fresh and activated SBTOC-F. (g(ii)) Oxygen content and photocatalytic superoxide radical production over fresh and activated SBTOC-F. (h) Absorption spectrum of SBTOC-F nanoplates correlated with wavelength-dependent AQY.

The H_2_O_2_ production efficiencies were investigated in the air by using pure water or water mixed with 10 vol% ethanol as a hole scavenger. In the presence of ethanol (condition (i), see the ESI†), SBTOC-S exhibited only a little H_2_O_2_ production as seen in Fig. 2a. SBTOC-F nanoplates, on the other hand, exhibited substantially enhanced activity, with an H_2_O_2_ evolution rate of ∼2.96 mmol h^−1^ g^−1^ (or ∼0.8 mmol h^−1^ m^−2^), nearly ∼3.3 times (or ∼1.45 times surface normalized) higher than that of SBTOC-S, confirming the rate-enhancing role of facet control beyond surface area improvement. The reactions were further performed employing different atmospheres to examine the active species responsible for H_2_O_2_ production. The production rate increases when the solution is saturated with oxygen (∼3.5 mmol h^−1^ g^−1^, Fig. 2b). Under nitrogen saturation, on the other hand, the production rate sharply decreases to ∼0.45 mmol h^−1^ g^−1^, suggesting that the formation of H_2_O_2_ occurs primarily *via* the 2e^−^ oxygen reduction reaction (ORR) pathway. We further confirmed this pathway by superoxide trapping experiments (Fig. S4†).^37^ These experiments revealed that photoirradiation of SBTOC-F produces excited hole (h^+^) and electron (e^−^) pairs. h^+^ oxidizes ethanol (C_2_H_5_OH + 2h^+^ → CH_3_CHO + 2H^+^), while e^−^ promotes the two-electron reduction of O_2_ to H_2_O_2_.^38^ In the process, the O_2_ reduction could occur following two distinct routes: a two-step single-electron O_2_ reduction (O_2_ + e^−^ → O_2_^˙−^ (−0.33 eV) and O_2_^˙−^ + e^−^ + 2H^+^ → H_2_O_2_ (1.44 eV)), or a direct two-electron O_2_ reduction (O_2_ + 2e^−^ + 2H^+^ → H_2_O_2_, 1.76 eV) route, both of which are feasible in SBTOC-F for its high C.B. position.^39^ Therefore, superoxide radical (O_2_^˙−^) formation was further quantified for both SBTOC-S and SBTOC-F using nitroblue tetrazolium in the air. As seen in Fig. 2c, the O_2_^˙−^ production over SBTOC-F (∼297 μmol h^−1^ g^−1^ or ∼82 μmol h^−1^ m^−2^) is significantly higher than that over SBTOC-S (∼66 μmol h^−1^ g^−1^ or ∼47 μmol h^−1^ m^−2^), loosely matching the H_2_O_2_ production rate in pure water in the air atmosphere (±11% deviation, discussed *vide infra*) and confirming the involvement of the two-step single-electron transfer route.

Importantly, the recyclability tests using SBTOC-S and SBTOC-F unfurled a rare self-activation phenomenon both in pure water and a water–ethanol mixture, unknown for any H_2_O_2_-producing photocatalyst so far. For example, when both of them were used for 5 consecutive days in pure water and an air atmosphere (condition (ii), see the ESI†), unexpectedly, the H_2_O_2_ production was found to increase consistently and significantly for both samples (Fig. 2d). This effect is similar in the water–ethanol mixture too and stands in contrast to typical photocatalysts, where catalytic activity progressively degrades within a few cycles. Additionally, the activity increment in SBTOC-F is ∼17 times more than that in SBTOC-S to establish the importance of facet control in (001) facet-exposed nanoplates.

To further evaluate, the recyclability of SBTOC-F nanoplates was extended up to 30 days (Fig. 2e) in pure water, and the activity kept on increasing from ∼590 μM (per 2 h) on the 1st day to ∼1470 μM (per 2 h) on the 21st day. Beyond this, the efficiency plateaued which is attributed to the oxidation of the catalyst by the *in situ* generated H_2_O_2_, hindering further surface vacancy formation responsible for self-activation, as discussed later. Fig. 2f shows the catalyst activity and activation over 15 days in a 10% ethanol–water solution (condition (i), see the ESI†). As seen by comparing with Fig. 2e, the extent of catalyst activation is similar in water as well as water–ethanol mixtures (∼two times), and therefore the activation process is not primarily linked to ethanol.

To comprehend the remarkable boost in activity, the capability of fresh and self-activated SBTOC-F nanoplates to photo-reduce molecular O_2_ to superoxide radicals was evaluated using the electron spin resonance (ESR) technique and spin-trapping with 5,5-dimethyl-pyrroline *N*-oxide (DMPO). Four characteristic DMPO–O_2_^˙−^ peaks were observed in both instances (Fig. 2g(i)), confirming the generation of O_2_^˙−^ and indicating a two-step single electron reduction process.^17,40,41^ In addition, the stronger signal intensity in the activated SBTOC-F than in the fresh nanoplates validates its enhanced O_2_^˙−^ generation activity. Spectral quantification showed that superoxide radical production also doubles in the activated sample to match the enhanced H_2_O_2_ production rate, indicating that the activated catalysts may adsorb more molecular O_2_ due to surface restructuring (discussed *vide infra*). We, therefore, estimated the total oxygen content in the catalyst solution and found it to be significantly high in the case of the activated sample (Fig. 2g(ii)).^42^ Notwithstanding surface reconstruction, the catalyst in bulk is highly stable as revealed by negligible changes in the powder XRD pattern of the used sample (Fig. S5a†).

The photocatalytic H_2_O_2_ peroxide production rate (>6 mmol h^−1^ g^−1^, Fig. 2f) by the SBTOC-F nanoplates is highly encouraging by considering that no noble metal or external oxygen was used, and no catalyst deactivation was observed (Table S1†). The wavelength-dependent apparent quantum yield (AQY) for the H_2_O_2_ production by SBTOC-F is seen in Fig. 2h, agreeing well with its absorption spectrum and exhibiting an AQY of 17.1% at 360 nm.

### 2.3 Origin of self-activation of SBTOC-F

**Fig. 3:**
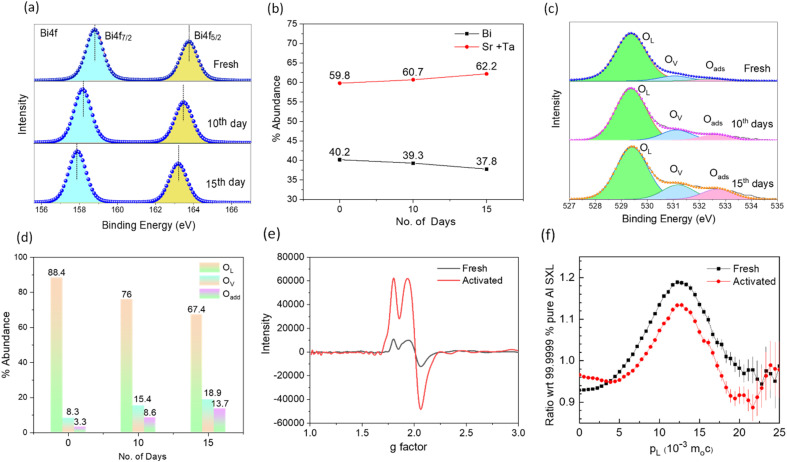
(a) Bi 4f XPS spectra of fresh and activated SBTOC-F. (b) Plot showing a decrease in the surface Bi content as compared to (Ta + Sr) in activated SBTOC-F. (c) High-resolution O 1s XPS spectra showing (d) an increase in oxygen vacancies and adsorbed oxygen in comparison to lattice oxygen in activated SBTOC-F. (e) EPR spectra of fresh and activated SBTOC-F. (f) Ratio curves generated from the coincidence Doppler broadening spectra of fresh and activated SBTOC-F.

The fundamental processes of surface reconstruction and molecular O_2_ activation, photon absorption, the exciton separation efficiency of the catalyst, *etc.*, were systematically examined to clarify the origin of the unusual increase in H_2_O_2_ production efficiency with increasing cycles. Noteworthy is that there were no discernible differences in the PXRD and surface area analysis of the samples before and after use (Fig. S5 and ESI Note 3†). However, X-ray photoelectron spectroscopy (XPS) indicated a significant surface reconstruction from vacancy generation processes. Fig. 3a compares the high-resolution XPS Bi 4f spectra of the fresh sample with those of the activated one showing an evident shift of ∼0.82 eV and 1.1 eV smaller binding energies after 10 and 15 days of activation, respectively, characterizing a change in the chemical environment of Bi atoms with a reduced number of coordinating oxygen atoms by oxygen vacancy (O_V_) formation.^43^ Recent theoretical studies revealed that O_V_ in metal oxides originate from the transfer of photogenerated electrons from the oxygen-contributed valence band to the bismuth-contributed conduction band and such reduced oxygen moieties can leave the surface as O_2_ molecules or –OH species.^43^ Alongside, the abundance of Bi also decreases (Fig. 3b) with respect to the total Sr and Ta content from 40.2% in fresh catalyst to 39.3% and 37.8% after 10 and 15 days of activation, respectively, indicating Bi leaching during reuse. The oxygen peaks were analyzed to affirm O_V_ formation. As seen in Fig. 3c, the O 1s XPS spectra are characterized by three peaks of lattice oxygen (O_L_, 529.3 eV), dissociative oxygen around Bi vacancies (O_V_, 531.0 eV), and surface-adsorbed molecular oxygen or hydroxyl species (O_ads_, 532.5 eV).^44^ The abundance of vacancy oxygen and adsorbed oxygen with respect to lattice oxygen increases in the activated sample (Fig. 3d) to confirm the leaching of lattice oxygen from the sample to facilitate molecular O_2_ adsorption.^45^ The Sr 3d and Ta 4f spectra, on the other hand, exhibited much fewer shifts (Fig. S6†), indicating that O_V_ formation in perovskite layers is less likely to take place during the activation of the catalyst, understandably due to a stronger (Sr/Ta)–O bond than Bi–O. Also, the stronger Lewis acidity of Bi as compared to Ta and Sr leads to easy formation of Bi–OH bonds, which precedes the leaching step.^45^ Similarly, we estimated the catalyst's Cl/Sr atomic ratio before and after activation which remains the same, implying that the dissolution of Cl under light irradiation is negligible. This we expected because the VBM of the Sillen–Aurivillus phase is composed of a dispersive O 2p band, while the Cl 3p orbital energetically lies far too below.^30^ For confirmation, the concentration of leached metal ions into the solution during the activation process was evaluated using inductively coupled plasma mass spectrometry (ICP-MS, Fig. S7†), which revealed that ∼11 parts per billion (ppb) of Bi per gram of catalyst leached from the nanoplates into the solution after a 10-day activation period, which increased to ∼16 ppb after 15 days. Leached Sr and Ta amounts were negligible. Also, it is to be noted that the solution containing the leached ions only (after removal of the catalyst by centrifugation) was found to have no photocatalytic activity towards H_2_O_2_ production or decomposition. Subsequently, the existence of O_V_ in the catalyst was confirmed by EPR spectroscopy (*g* = 2.003, Fig. 3e).^17,46,47^ A threefold increase in EPR peak intensity in the activated catalyst asserts a high level of oxygen vacancies.

Positron annihilation lifetime (PAL) and coincidence Doppler broadening (CDB) spectroscopies were employed to characterize the chemical nature of the defects. PAL spectra were fitted with three lifetime components (Table 1); the longer component (*τ*_3_) ∼2300 ps with small intensity (∼2%) corresponds to the pick-off annihilation of the positronium atoms. *τ*_1_ and *τ*_2_ are attributed to isolated vacancies and Bi–O vacancy associates respectively, in the form of 
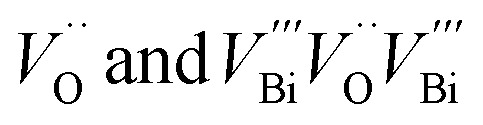
.^48–50^ The relatively high contribution of *τ*_2_ in both the fresh and activated samples suggests that Bi–O vacancy associates are the predominant forms of defects in the Sr_2_Bi_3_Ta_2_O_11_Cl nanoplates. Additionally, the relative intensity of *τ*_2_ further increases with activation whereas *τ*_1_ decreases to depict that the isolated vacancies are progressively converted into vacancy associates during reuse, leading to a non-linear activity increase (ESI Note 4†). CDB spectra were analyzed by taking a point-by-point intensity ratio with a 99.9999% pure aluminum single crystal. The ratio curve of CDB of the fresh and activated samples (Fig. 3f) shows a characteristic peak at p_L_ = 11 × 10^−3^ × *m*_0_*c*, attributed to the annihilation of positrons with the 2p electrons of oxygen anions (*m*_0_: electron rest mass and *c*: speed of light).^51^ The decreased peak intensity with the activation confirms an increase in the number of vacancy clusters by decreasing the oxygen anions.

**Table 1:** Positron lifetime parameters of the fresh and activated SBTOC-F nanoplates

Sample	*τ*_1_ (ps)	*I*_1_ (%)	*τ*_2_ (ps)	*I*_2_ (%)	*τ*_3_ (ps)	*I*_3_ (%)
Fresh	105 ± 3	31 ± 2	295 ± 2	67 ± 2	2218 ± 51	2 ± 0.1
Activated	107 ± 3	25 ± 2	299 ± 5	73 ± 2	2306 ± 83	2 ± 0.1

**Fig. 4:**
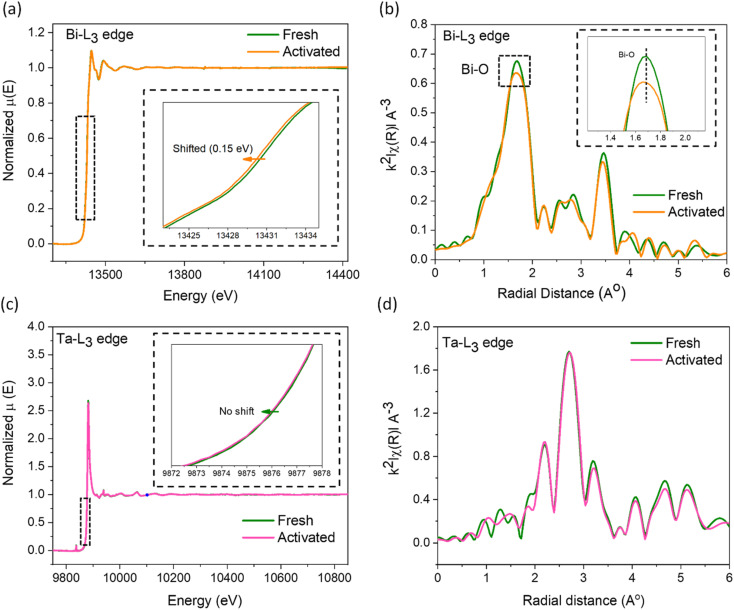
(a) Bi-L_3_ edge XANES and (b) EXAFS spectra of fresh and activated SBTOC-F. (c) Ta-L_3_ edge XANES and (b) EXAFS spectra of fresh and activated SBTOC-F.

X-ray absorption spectroscopy studies were conducted on the fresh and the used catalysts to visualize the electronic and geometric structural distortions arising from increased defect concentrations. The X-ray absorption near edge spectra (XANES) of the Bi-L_3_ edge for the activated catalyst moves to a lower binding energy (∼0.15 eV, Fig. 4a), indicating an average reduced oxidation state of bismuth arising from the surrounding oxygen vacancies, corroborating the d-orbital shifts in the XPS analysis. In addition, the smaller energy shift while using the high-energy synchrotron beam infers the nanoplate surface as the primary region for O_V_ creation. From the extended X-ray absorption fine structure spectroscopy (EXAFS, Fig. 4b), we observed a major peak at the *k*^2^-weighted oscillations in the *R*-space centering ∼1.7 Å corresponding to the Bi–O bond.^52,53^ The reduction in its intensity by ∼6% as well as its shift towards lower *R* confirms the decrease in the oxygen coordination number around the Bi atoms and a distorted local geometry in the Bi_2_O_2_ layer.^50,54^ Conversely, negligible changes were observed for the Ta-L_3_ edge in XANES and EXAFS data (Fig. 4c and d), suggesting the higher stability of the Ta–O perovskite layer originating from the higher Ta–O bond strength. Earlier studies on oxide phases undergoing surface reconstruction induced by ion leaching revealed the formation of surface amorphous layers.^55^ Therefore, we further examined the fresh and activated samples using TEM. As shown in Fig. S8,† crystalline particle edges in the fresh catalyst transform into an amorphous layer after activation, reaching a depth of 0.2–2 nm in some particles, accompanied by the emergence of a heightened number of defects at the interface between the crystalline and amorphous phases in the activated catalyst. Overall, the detailed XRD, EPR, XPS, PAS, EXAFS, ICP-MS, and TEM analyses of the fresh and the activated samples confirmed that the surfaces of the nanoplates reconstruct under the experimental conditions from preferential leaching of Bi and O atoms to form an amorphous surface layer.

The diverse sets of experiments strongly support the notion that defects in SBTOC-F serve as active sites for molecular oxygen adsorption that transforms into H_2_O_2_, as shown earlier,^43^ and the increase of which leads to self-activation. The self-activation plateaued after 21 days, attributed to compensation for further defect formation by their *in situ* annihilation by H_2_O_2_. To confirm this, we subjected the catalysts at various stages of activation to concentrated H_2_O_2_ treatment at room temperature, with the intention of reducing the O_V_ concentration. This resulted in a significant decrease in activity for all of them (Fig. S9 and S10† for the fresh catalyst for example).

**Fig. 5:**
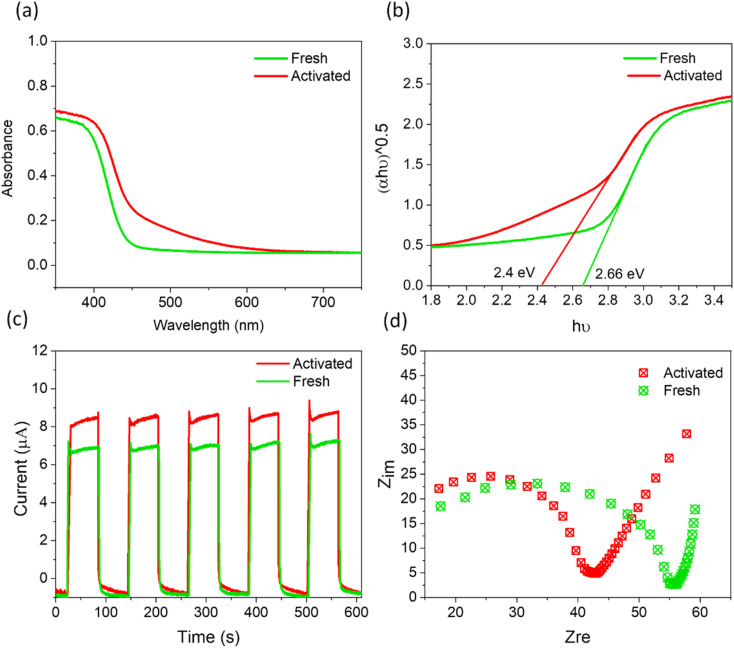
(a) DRS and (b) the corresponding Tauc plots, (c) transient photocurrent response, and (d) Nyquist plots for fresh and activated SBTOC-F.

Beyond the increasingly efficient harvesting of molecular O_2_ using SBTOC-F, several additional factors contributed to the self-activation of the catalyst. These include superior charge separation efficiency, improved light absorption, and enhanced electron transfer properties. Metal-rich vacancy-containing surfaces usually create shallow trap states that can improve light harvesting and also charge transfer. In a Sillen phase, the presence of Bi and oxygen vacancies was recently shown to shift the valence band for enhanced light absorption as well as transfer rates of photogenerated charges to reactants, leading to an order of magnitude increase in photocatalytic activity.^56,57^ The UV-vis DRS plots (Fig. 5a and b) of our activated samples also showed a similar shift of the optical absorption edge to longer wavelengths, leading to a narrower band gap of 2.4 eV. To investigate its effect on their ability to separate and transport the photogenerated charge carriers, we further carried out photocurrent and electrochemical impedance spectroscopic measurements. As shown in Fig. 5c, both the fresh and the activated catalysts were quick to produce photocurrent in response to the visible light. However, the activated nanoplates displayed a higher photocurrent response than the fresh one, indicating more effective charge separation in it, attributed to the trapping of excited electrons at the shallow vacancy states.^15^ In addition, electrochemical impedance spectroscopy (Nyquist plot, Fig. 5d) revealed that the electron-transfer resistance (*R*_ct_) of activated nanoplates is ∼20% lower than that of fresh nanoplates, implying a low charge transfer resistance originating from the low valent metal moieties.

Thus, in brief, excess molecular oxygen harvesting, superior charge separation efficiency, improved light absorption, and electron transfer properties contribute to high catalytic activity by SBTOC-F and further self-activation upon its recycling (Fig. S11†).

## 3 Conclusion

In summary, we show that during facile photocatalytic H_2_O_2_ production, the competitive leaching of metal ions and anions within a complex inorganic lattice framework can overcompensate for catalyst degradation observed in the usual photoactive materials, and this can continuously improve the efficiency of an already facile photocatalyst. Our single-crystalline Sr_2_Bi_3_Ta_2_O_11_Cl nanoplates enclosed by the [100], [010], and [001] facets have triple the surface area that is achievable by traditional methods and a facile H_2_O_2_ production rate of ∼2.9 mmol h^−1^ g^−1^ in the air. Furthermore, the progressive leaching of surface Bi and oxide anions generates more surface vacancies that transform into vacancy clusters and aid in excess O_2_ adsorption. The shallow energy levels of the vacancies lead to excess light absorption and facile exciton separation to further improve catalytic efficiency synergistically, leading to doubled H_2_O_2_ production in 15 days. The findings establish the potential of rational defect engineering in developing sustainable photocatalysts for realistic applications by avoiding catalyst deactivation.

## Data availability

The data supporting this article have been included as part of the ESI.†

## Author contributions

U. K. G. supervised the research. U. K. G. and M. B. designed the experiments. M. B., A. K. S., R. S. R., K. K., and B. K. contributed to performing the catalytic experiments, EXAFS, and XPS characterization. D. S. performed positron annihilation measurements. M. B. did material characterization. All authors discussed the results and commented on the manuscript.

## Conflicts of interest

The authors declare no conflict of interest.

## Supplementary Material

SC-OLF-D4SC04866K-s001
