# Supplementary material for: A surface reconstruction route for increasingly improved photocatalytic H2O2 production using Sr2Bi3Ta2O11Cl
Source: Chem Sci. 2024 Sep 18;15(41):17049–57. doi: 10.1039/d4sc04866k (PMC11423514; doi:10.1039/d4sc04866k)
Supplement: SC-015-D4SC04866K-s001 [file SC-015-D4SC04866K-s001.pdf]

Supplementary Information (SI) for Chemical Science.
This journal is © The Royal Society of Chemistry 2024

Supplementary Information

Surface Reconstruction Route for Increasingly Improved Photocatalytic H_2O_2 Production Using $Sr_2Bi_3Ta_2O_{11}Cl$

Maqsuma Banoo,[1] Arjun Kumar Sah,[1] Raj Sekhar Roy,[1] Komalpreet Kaur,[1] Bramhaiah Kommula,[1] Dirtha Sanyal,[2,3] Ujjal K. Gautam[1*]

[1]Department of Chemical Sciences, Indian Institute of Science Education and Research (IISER)-Mohali, Sector 81, Mohali, SAS Nagar, Punjab 140306, India

[2]Variable Energy Cyclotron Centre, 1/AF Bidhannagar, Kolkata 700064, India

[3]Homi Bhabha National Institute, Anushakti Nagar, Mumbai 400094, India

**Corresponding author: ujjalgautam@iisermohali.ac.in, ujjalgautam@gmail.com*

This file includes:

Supplemental experimental section

Supplemental scheme (Scheme 1)

Supplemental Note (Note 1-2)

Supplemental Figures (Figures S1 to S10)

Supplemental AQY Calculation

Supplemental table (Table S1).

Supplemental references

1. Supplemental Experimental Section

1.1. Characterization

Powder X-ray diffraction (XRD) patterns were recorded using a Rigaku Ultima IV diffractometer equipped with Cu Kα X-ray radiation with a wavelength of 1.5406 A° (40 kV and 40 mA). The data were collected from 10° to 70° over 2θ with a scanning speed of 1degree/minute in 0.02 steps. The morphology of the samples was analyzed by field-emission scanning electron microscope (FESEM, JEOL, JSM-7600F, accelerating voltage of 20-30 kV), transmission electron microscope (TEM, JEOL JEM-F200), and atomic force microscope (AFM, Park System XE7). SEM and AFM samples were prepared by drop-casting ethanolic dispersions of the samples on silicon wafers and allowing the solvent to evaporate slowly at room temperature in a vacuum desiccator. Ethanolic dispersion of the sample was drop-casted on a carbon-coated Cu grid for TEM observation. AXIS ULTRA XPS spectrometer was used for X-ray photoelectron spectroscopy measurements. BET surface area was recorded on a Quantachrome instrument (Autosorb-Iq-c-XR-XR). The apparent surface area was calculated by the Bruner Emmett Teller (BET) method. UV-Vis spectrophotometer (Cary 5000), using $BaSO_4$ powder as the reference was used to record diffuse reflectance spectra (DRS). Perkin Elmer Lambda 365 spectrophotometer was used to record UV-vis absorption spectra at room temperature. Electron paramagnetic resonance (EPR) measurements were carried out on a Bruker A300-9.5/12/S/W spectrometer at room temperature operating with a microwave

frequency of 9.75 GHz. The active radical species were trapped by adding 5,5-dimethyl-1-pyrroline N-oxide (DMPO) as a spin-trapping reagent. Typically, the experiments for superoxide radicals were conducted in a water-ethanol solution. Then the solution was irradiated with a light source while stirring for 30 min. The resulting solution was subjected to analysis at room temperature in a sealed capillary tube after the addition of DMPO. Inductively coupled plasma mass spectrometry (ICP-MS) was performed (Agilent7900) to determine the metal content leached into the solution.

Positron annihilation lifetime (PAL) has been measured with a pair of gamma-ray detectors (ultrafast 2.5 cm × 2.5 cm BaF_2 scintillator optically coupled with XP2020Q photomultiplier tube) based **γ-γ** coincidence (fast-fast) assembly. The details of the positron annihilation lifetime setup are described elsewhere.[1,2] The source of the positron is 8 µCi (micro-Curie) ^{22}NaCl deposited in a 6 µm thick mylar foil covered by another 6µm thick mylar foil. PATFIT-88 computer program has been used to deconvolute the recorded PAL spectrum with proper source corrections.[3] The source components have been estimated by taking the positron lifetime spectrum of 99.9999% aluminum sample and also with a pure silicon sample. The estimated absolute source contribution is about 5%. The absolute source contribution is subtracted in the "positron-fit" program for estimating the positron lifetime components in the sample.

For the Coincidence Doppler broadening (CDB) studies, a pair of HPGe detectors of 12% efficiency (model number PGC 1216sp of DSG, Germany) has been used. The detectors are placed at 180 ^{o} angles centering the source sample sandwich. The energy resolution of this HPGe detector is about 1.15 keV at 514 keV of 85 Sr. The Doppler broadening data have been recorded in a dual ADC-based multiparameter data acquisition system (Model # MPA-3 of FAST ComTec, Germany). The coincidence Doppler broadening spectra have been analyzed by taking the area normalized point-by-point ratio with a CDB spectrum of 99.9999 % pure aluminum single crystal.

X-ray absorption near-edge spectroscopy (XANES) and extended X-ray absorption fine structure (EXAFS) experiments at 300 K were performed at PETRA III, beamline P64, DESY, Germany. Measurements of Bi-L_3 and Ta-L_3 edges at ambient pressure were performed in fluorescence mode. Monochromatic X-rays were obtained using a Si (111) double-crystal monochromator. The acquired XAFS raw data was processed for background subtraction, normalization, and Fourier transformations using ATHENA software.

The electrochemical measurements were performed by using an electrochemical analyzer (CHI760E electrochemical workstation) adopting a three-electrode configuration. Ag/AgCl (saturated KCl) was used as a reference electrode, Pt wire as a counter electrode, and ITO as the working electrode. A typical working electrode was prepared by dispersing 5 mg of the sample in 400 μL of a mixed solution of isopropyl alcohol, Nafion, and water by sonication. Afterward, the resulting suspension was drop-casted on an ITO glass and exposed to air overnight to dry. The electrolyte was an aqueous solution of (0.2 M) Na_2SO_4. The Mott-Schottky plot was obtained by measuring the capacitance as a function of potentials at two distinct frequencies, 200 Hz and 800 Hz in the potential range from −0.8 to 0 V. Photocurrent response was measured by linear sweep voltammetry at a scan rate of 10 mV s^{-1}. 400 W Xe-lamp was used to illuminate the electrode. Electrochemical impedance spectroscopy (EIS) tests were performed at open circuit potential over a frequency range of between 0.1 and 10^6 Hz, with an AC voltage magnitude of 5 mV.

1.2 Experimental Procedures

1.2.1. Materials. Ta_2O_5 (Sigma Aldrich, 99.9%), $SrCO_3$ (HIMEDIA, Ltd., 99.5%), BiOCl (HIMEDIA, Ltd., 99.5%), and Bi_2O_3 (HIMEDIA, 99.99%) as raw materials. Alkali metal chloride (KCl (HIMEDIA, 99.5%), and NaCl (HIMEDIA, 99.5%) (NaCl/KCl) as a flux. K.I. (Sigma Aldrich, 99.8%), $H_{32}Mo_7N_6O_{28}$ (Sigma Aldrich, 99%), Nitroblue tetrazolium (Sigma Aldrich, 90%), and 5,5-dimethyl-pyrroline N-oxide (DMPO, G.L.R., 98%) for (Reactive Oxygen Species) R.O.S. detection were used without further purification.

1.2.2. Flux Synthesis of $Sr_2Bi_3Ta_2O_{11}Cl$: $Sr_2Bi_3Ta_2O_{11}Cl$ nanoplates were synthesized by employing a stoichiometric molar ratio (2:1:1:1) of $SrCO_3$, Bi_2O_3, Ta_2O_5, and BiOCl as precursors. To facilitate the reaction and ensure nanoplate formation, a molten salt composed of a eutectic mixture of KCl and NaCl (1:1) was utilized as a flux. The solute concentration of the flux was maintained at 3.2 mol%, relative to the total amount of $Sr_2Bi_3Ta_2O_{11}Cl$ and flux. The precursors and flux were meticulously ground using a mortar and pestle, placed in an aluminum crucible, and heated at 1023K for 4 hours in a muffle furnace. After natural cooling, the product was washed with deionized water, collected through filtration, and the resulting off-white powder was dried at room temperature. For comparison, $Sr_2Bi_3Ta_2O_{11}Cl$ is also prepared by the traditional solid-state method.

1.2.3. Solid state synthesis of $Sr_2Bi_3Ta_2O_{11}Cl$: $Sr_2Bi_3Ta_2O_{11}Cl$ agglomerated grains were synthesized by a two-step procedure. $SrBi_2Ta_2O_9$ and $SrBiO_2Cl$ were first prepared separately.

$SrBi_2Ta_2O_9$ was prepared by using a stoichiometric mixture of Bi_2O_3, Ta_2O_5, and $SrCO_3$ and heated at 1073 K for 5 h in air. $SrBiO_2Cl$ was also synthesized by calcination of a mixture of BiOCl (10 mmol) and $SrCO_3$ (10 mmol) at 1073 K for 10 h in air. Finally, the main target compound was synthesized by heating the $SrBi_2Ta_2O_9$ and $SrBiO_2Cl$ precursors. $SrBi_2Ta_2O_9$ (1.0 mmol) was thoroughly mixed with $SrBiO_2Cl$ (1.05 mmol) and heated in a muffle furnace at 1123 K for 15 h in air. Both procedures are summarized in Scheme-1

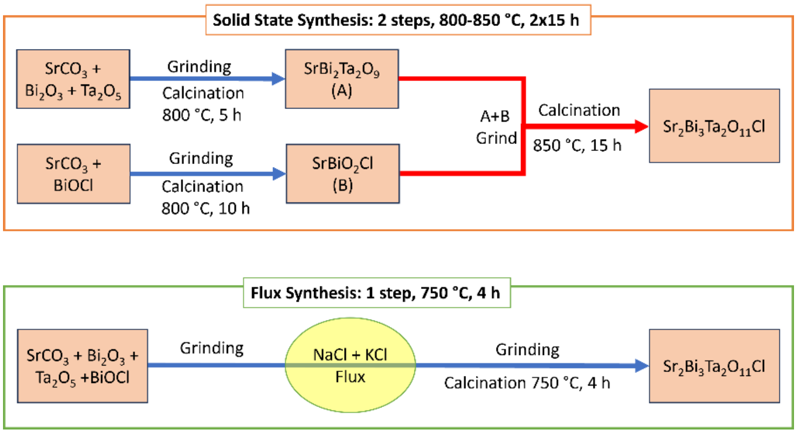

Scheme 1: Synthesis procedure of $Sr_2Bi_3Ta_2O_{11}Cl$ by flux method and Two-step solid states synthesis method.

Supplemental Note 1 (Advantages of using Flux synthesis): It has emerged from recent studies and our controlled experiments, discussed below, that using a eutectic mixture of NaCl and KCl is advantageous for several reasons. First, the melting points of NaCl and KCl are higher than 1023 K (750 °C). Therefore, using the mixture reduces the melting point of the flux and the synthesis can be carried out at a lower temperature. Second, BiOCl is somewhat volatile and several recent reports emphasize the usefulness of using flux to avoid halide vacancy.[4] Third, it is also reported that flux increases the reaction rate by increasing the diffusion rate of the reactants.[5] Fourth, it is also widely reported that the use of flux leads to highly crystalline products with facet control.[6]

We observed significant advantages while using flux synthesis because it reduced the reaction time to 4 hours and a single step unlike in the solid-state synthesis which involves two steps and takes a total of about 30 hours of heating. Besides, we also observed a higher (more than twice) surface area for the flux sample than the solid-state synthesized sample which is important for heterogeneous catalysis.

It was also reported recently that the halide component is contributed by the flux used.[4] We followed the same procedure to confirm a similar mechanism and synthesized $Sr_2Bi_3Ta_2O_{11}Cl$ by replacing the chloride flux (NaCl/KCl) with bromide flux (NaBr/KBr). From PXRD (Figure S1a), we could ascertain that there are systematic shifts of the diffraction peaks to lower 2theta values due to the presence of larger Br atoms. However, all peaks were retained for the new compound suggesting an impurity-free compound. We also analysed the sample using TEM-EDS as shown in Figure S1b. The EDS analysis shows barely any presence of chloride while the presence of bromide is overwhelming. This confirms that the halide component is contributed from the flux.

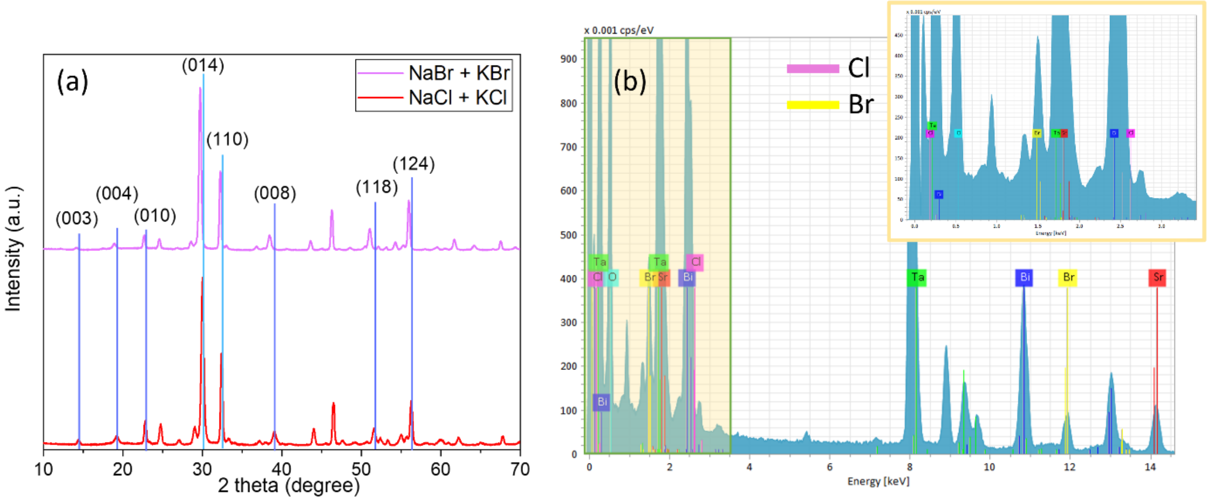

Figure S1: (a) Powder XRD pattern of $Sr_2Bi_3Ta_2O_{11}Cl$ with NaCl/KCl and NaBr/KBr Flux. (b) TEM-EDS of $Sr_2Bi_3Ta_2O_{11}Cl$ synthesis with NaBr/KBr Flux.

Supplemental Note 2: To evaluate preferred and restricted growth orientations in a nanoplate of SBTOC-F, we compared specific peak intensities in the experimental and simulated pattern. The simulated pattern was created using the .cif file provided by *S. Liu et al*[7] and replacing Br with Cl, and then further refining the lattice parameters.

From TEM analysis, we propose that the growth is restricted in the [001] direction in comparison to the [100] and [010] directions, and therefore the diffraction peak intensity ratios of (00z) peaks to (xy0) are expected to be lower in the experimental pattern in comparison to the simulated one. Indeed, the peak intensity ratio for (003)/(110) which is just 0.27 in the simulated pattern but 0.62 in the experimental pattern. This confirms that the growth along the

(001) direction is less in the nanoplates as compared to (100) or (010) directions, which complies with the TEM observation discussed later in the manuscript. A model of the nanoplate showing the growth direction is presented in Figure S2d.

1.2.4. Photocatalytic reactions: The photocatalytic reactions were performed under two different conditions:

Condition (i): A 50 ml glass beaker and a 400 W Xe lamp with a light intensity of 128 mW/cm^2 were used as a light source in a top-down irradiation setup. Here, 10 mg of the as-synthesized or activated photocatalyst was dispersed in 30 mL ethanol aqueous solution (10 vol%) and then exposed to light. 500 µl of an aliquot from the reaction solution was taken after each 20 min interval and treated with 2 ml 0.1 M KI solution and 50 µl 0.01 M $H_{32}Mo_7N_6O_{28}$ solution. The data at 20 min was used in all experiments to determine the H_2O_2 formation rate since at longer time intervals, the back reaction becomes prominent. The concentration of H_2O_2 was obtained by evaluating the absorbance of the KI/ $H_{32}Mo_7N_6O_{28}$ combination at 352 nm by UV-Vis spectroscopy.

For recyclability, 200 mg of catalyst was dispersed in 200 ml of (10 vol%) ethanol aqueous solution and kept under natural sunlight in a 250 ml beaker. At the end of the day's sunlight exposure, the solution was removed by centrifugation to remove leached-out ions and H_2O_2 generated during the day, then fresh water was added to the beaker and exposed to sunlight the next day again. After the desired period of exposure (say 2 or 3 days), 10 mg of the catalyst was taken out of it and used to evaluate the H_2O_2 production rate using 30 mL ethanol aqueous solution (10 vol%) and 400 W Xe lamp.

Condition (ii): 10 mg of the as-synthesized photocatalyst was dispersed in 30 mL of pure water and then exposed to natural sunlight at the institute campus when the sunlight was sufficiently bright (power density: 100±10 mW/cm^2 and ~23% within the day). The concentration of H2O2 was detected by the same procedure as in condition (i).

For recyclability, 200 mg of catalyst was dispersed in 200 ml of pure water and kept under natural sunlight in a 250 ml beaker. At the end of the day's sunlight exposure, the solution was removed by centrifugation to remove leached-out ions and H_2O_2 generated during the day, then fresh water was added to the beaker and exposed to sunlight the next day again. After the

desired period of exposure (say 2 or 3 days), 10 mg of the catalyst was taken out of it and used to evaluate the H_2O_2 production rate using 30 mL of pure water and sunlight.

We noted that the catalysts are highly stable when stored in air. The reproducibility of H_2O_2 production using the same catalyst stored in air for nearly a year is within 5-7% error.

1.2.5. Detection of superoxide radicals: The experiment was performed by dispersing 40 mg of $Sr_2Bi_3Ta_2O_{11}Cl$ nanoplates into 25 mL of nitro blue tetrazolium (NBT) (0.025×10^{-3} m) solution, and then the reaction mixture was exposed to light irradiation for 3 h using a 400 W Xe lamp. The suspension was analyzed by UV-Vis absorption spectroscopy after the removal of the solid catalyst by centrifugation.

1.2.6. Measurement of total oxygen content in the aqueous dispersion of a catalyst: Non-invasive Ocean Optics Neofox-Kit-Probe was used to measure the total oxygen (TO) content in pure water and an aqueous dispersion of fresh and activated $Sr_2Bi_3Ta_2O_{11}Cl$ nanoplates at room temperature (25-27 ^{o} C).[8] Typically, the experiment was done by attaching the sensing patch to the inner wall of the 50 ml round bottom flask in such a way that it is immersed in the aqueous solution and measures the amount of oxygen dissolved in it. First, the amount of oxygen dissolved in pure water was measured, which was 322 µmol/L. Then to check the effect of the catalyst on oxygen content, 10 mg of $Sr_2Bi_3Ta_2O_{11}Cl$ nanoplates (fresh/activated) was dispersed in 100 ml of water.

1.2.7. Superoxide Radical Trapping Experiment: The Superoxide Radical trapping experiments were done by using 1.5 ml (10-2g/mol) benzoquinone (BQ) solutions and added into the reaction system i.e. 30 ml of water having 10 mg of catalyst. The reaction system was subjected to continuous irradiation of light. The H_2O_2 production was analyzed by analyzing the absorbance at 352 nm by UV-Vis. spectroscopy.

2. Supplemental Figures

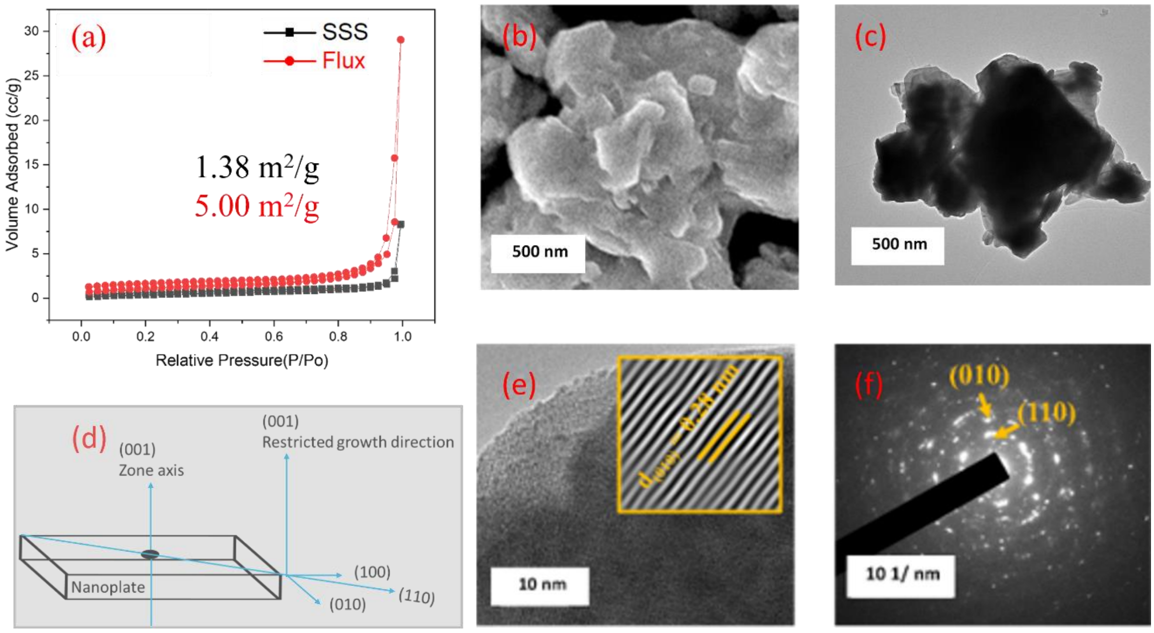

Figure S2: (a) BET adsorption isotherms of SBTOC-F and SBTOC-S (b,d,e,f) SEM, TEM, HRTEM images and SAED pattern of SBTOC-S. (c) A schematic showing the different facets of a SBTOC-F nanoplate and its restricted growth direction.

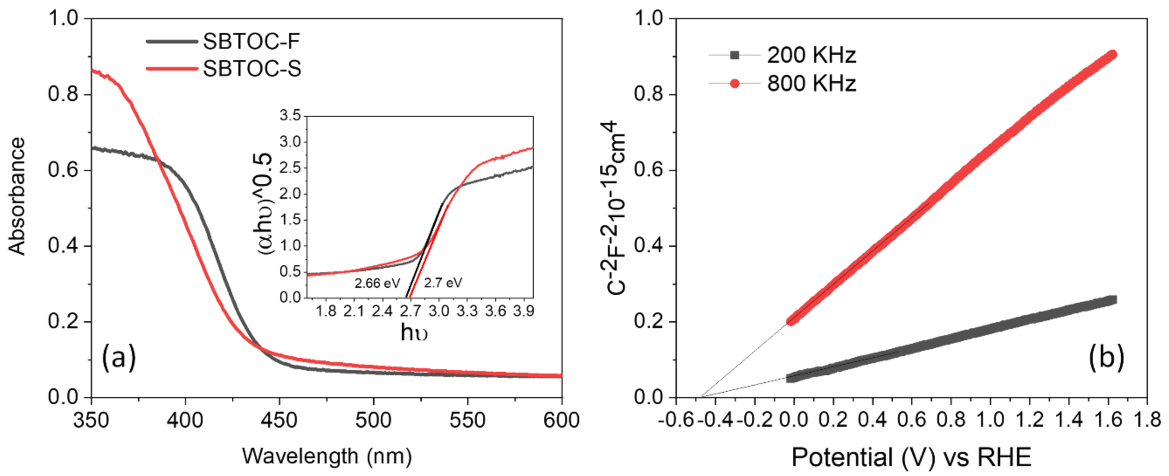

Figure S3: (a) UV-Vis diffuse reflectance spectra of SBTOC-S and SBTOC-F (inset is the corresponding Tauc Plot). (b) Mott–Schottky plots of SBTOC-F measured at 200 and 800 Hz.

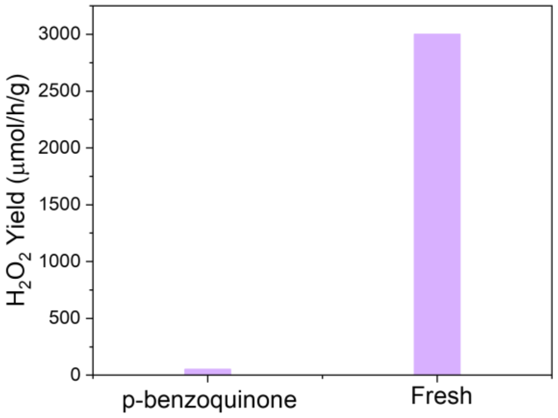

Figure S4: Plot showing the effect of superoxide radical trapping agents on H_2O_2 production.

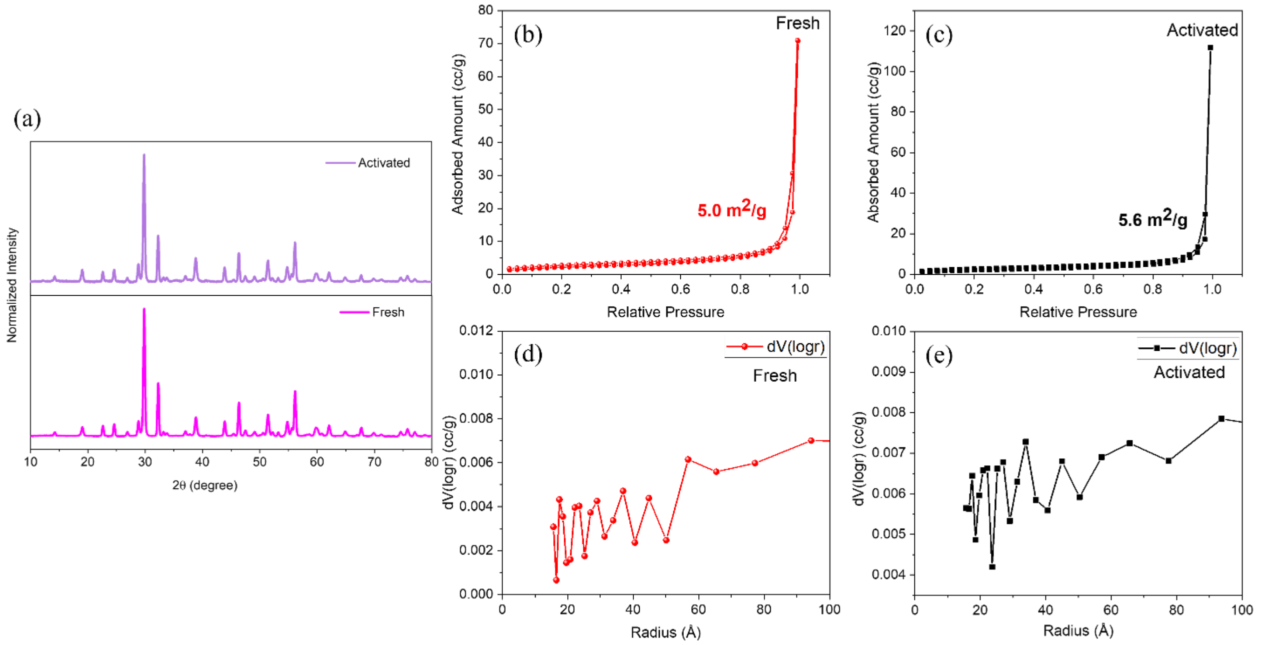

Figure S5: (a) Powder XRD patterns of $Sr_2Bi_3TaO_{11}Cl$ nanoplates before and after catalysis. N2 adsorption isotherms for the (b) fresh and (c) activated $Sr_2Bi_3Ta_2O_{11}Cl$, (d) and (e) are the corresponding BJH size distribution plots.

(**Supplemental Note 3 on Figure S5b-e**: To assess the impact of the self-activation process on the catalyst, we conducted BET measurements and pore structure analysis on the used catalyst. It indicated that there is negligible change in the surface area after self-activation. Additionally, pore size distribution analysis showed that the material lacks significant porosity, which is expected given its crystalline nature from high temperature flux synthesis, as confirmed by PXRD and SAED data. The average pore volumes and pore sizes, respectively, are 0.0161, 0.0169 cc/g, and 66, 56 Å for the fresh and activated samples, which are practically the same.)

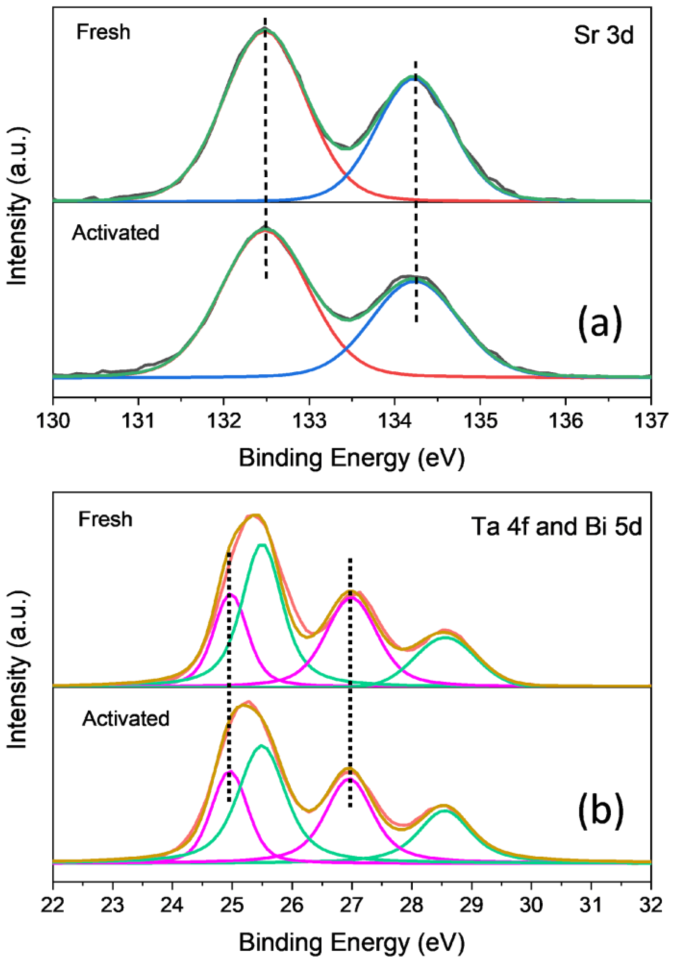

Figure S6: (a) High-resolution XPS spectra of Sr-3*d* and (b) Ta-4*f* in fresh and activated SBTOC-F.

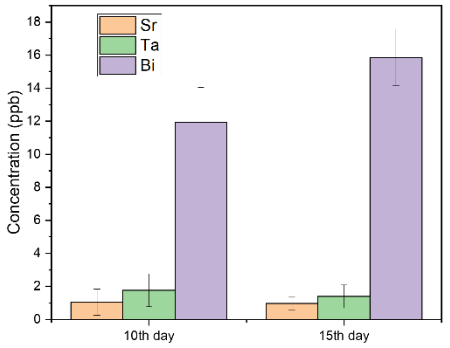

Figure S7: ICP-Ms data showing increases in Bi leaching from nanoplate to the reaction solution with increasing activation day.

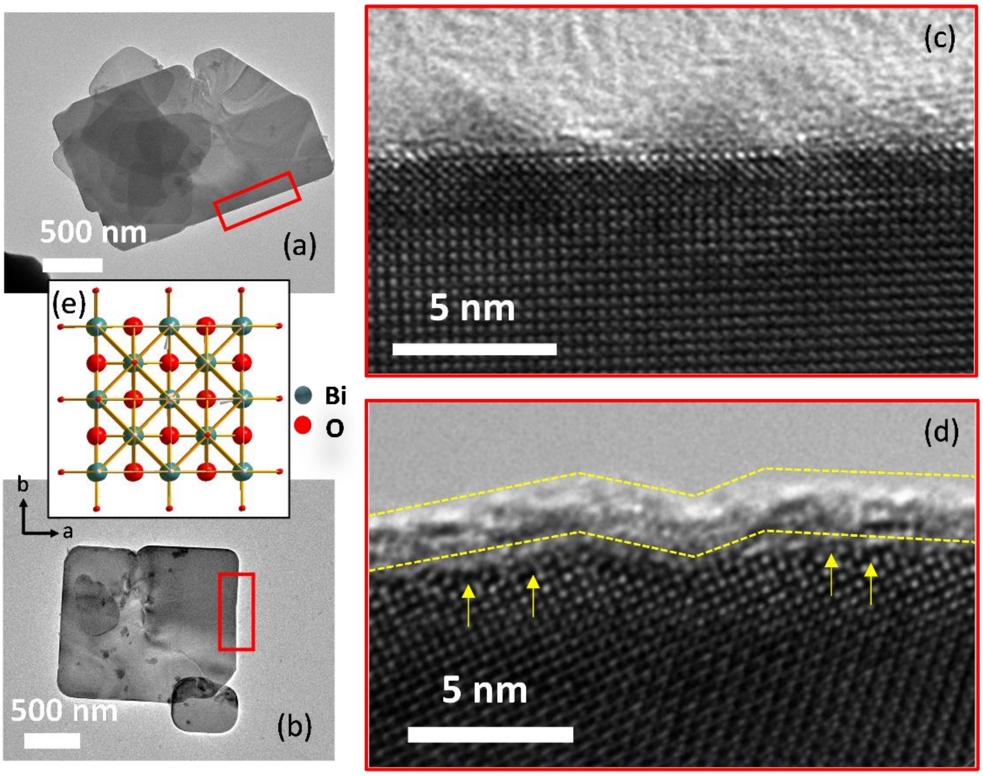

Figure S8: (a and b) TEM and (c and d) HR-TEM image of SBTOC-F nanoplates before and after catalysis respectively. (e) Crystal structure of SBTOC-F nanoplate along the ab plane as seen in HRTEM images.

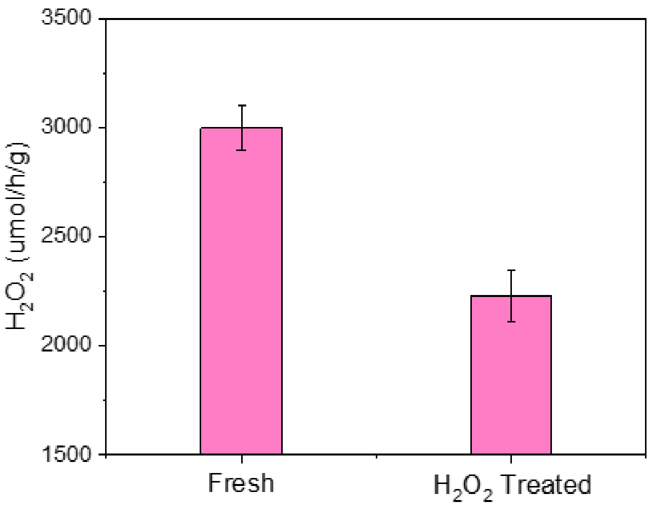

Figure S9: Plot showing the decreases in H_2O_2 production with decreasing the oxygen vacancy concentration by oxidizing the sample with H_2O_2.

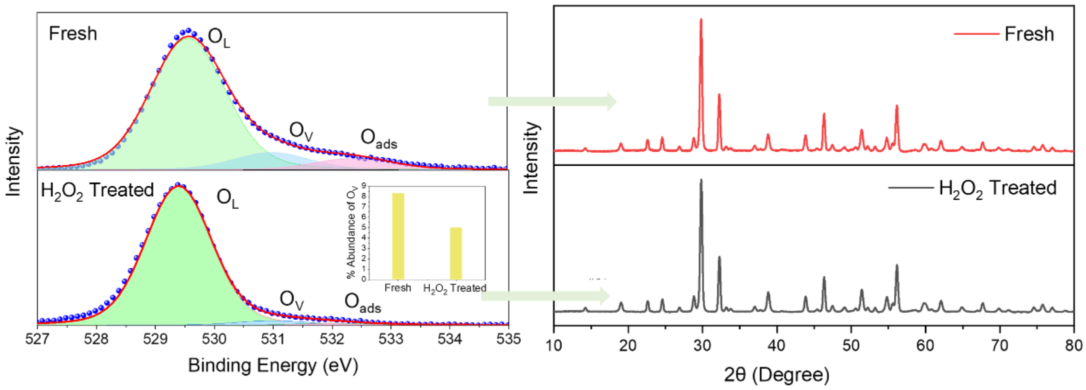

Figure S10: (left penal) High-resolution O-1s XPS spectra of fresh and H_2O_2 treated $Sr_2Bi_3Ta_2O_{11}Cl$ nanoplates confirming the decreases in oxygen vacancy concentration (inset: % abundance of oxygen vacancy with respect to lattice oxygen and surface adsorbed oxygen). (right penal) PXRD patterns showing the stability of $Sr_2Bi_3Ta_2O_{11}Cl$ at bulk level after H_2O_2 treatment.

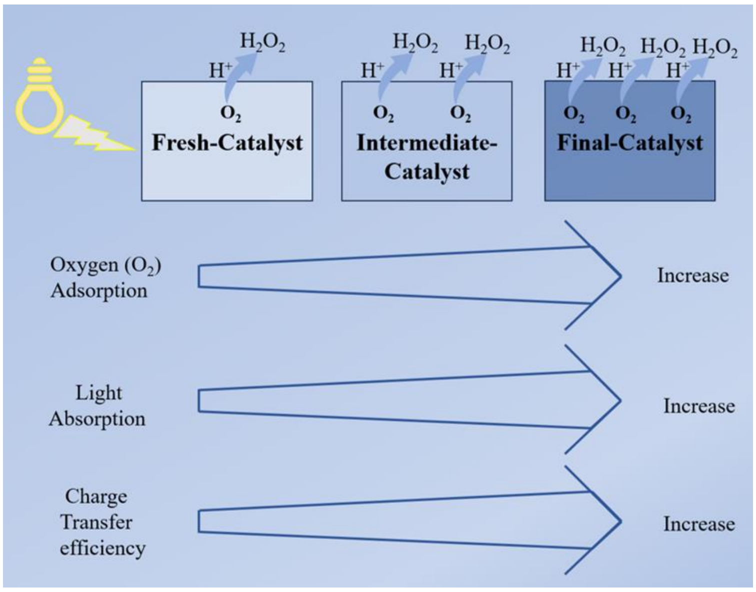

Figure S11: Schematic showing that during self-activation by defect formation, O_2 adsorption, light-absorption by the catalyst and charge transfer from catalyst to reactant improves continuously.

3. Calculation of Apparent Quantum Yield: The apparent quantum yield (AQE) for photocatalytic H_2O_2 production over flux synthesized $Sr_2Bi_3Ta_2O_{11}Cl$ nanoplate was calculated by carrying out the reaction under a 400 W Xe lamp as a function of wavelength with the help of different bandpass filters. (Newport, Model No.- 843-R) attached with a thermopile sensor (Newport, Model No.- 919P-010-16) was used to measure the power of the incident beam ($P_{incident}$) coming through a particular bandpass filter. Incident power density on the sample corresponding to a photon of wavelength λ ($\rho_{incident}$) was calculated as;

$$P_{incident} = \rho_{incident(\lambda)} \times A_{sample}$$

where A_{sample} is the area exposed to the incident light (20 cm^2).

The number of moles of incident photons per second $N_{ph(\lambda)}$, as a function of wavelength can be expressed as:

$$N_{ph(\lambda)} = \frac{\rho_{incident(\lambda)}}{N_A E_{ph(\lambda)}}$$

where $\rho_{incident(\lambda)}$ is the incident power density on the sample and N_A is the Avogadro number and $E_{ph(\lambda)}$ (= hc/λ) is the energy of one photon for the corresponding wavelength.

To correlate with the number of moles of hydrogen peroxide produced per hour, the total number of moles of the incident photon was calculated using the equation given below:

number of moles of incident photons per hour = $N_{ph(\lambda)} \times 3600 \times$ area of an incident beam.

Finally, the AQE can be derived from the following equation:

$$AQE = \frac{n\ (No.of\ electron\ transferred) \times Number\ of\ moles\ of\ hydrogen\ peroxide\ produced\ per\ hour}{Total\ no.of\ moles\ of\ incident\ photons\ per\ hour} * 100\%$$

where n=2 for hydrogen peroxide production

Supplemental Note 4 on non-linear activity rise during self-activation: The nonlinear increases in catalytic activity within 30 days is interesting and we attribute it to the type and dynamics of vacancy formation on the catalyst surface that drives the self-activation process.

We have multiple vacancy types as indicated by PAS studies, such as isolated oxygen, bismuth vacancies, etc. and as time progresses, we observe the formation of vacancy clusters. These vacancies heightened the molecular O_2 adsorption capability of the catalyst with time. This process, in turn, leads to an increased concentration of superoxide radicals, an intermediate in the formation of H_2O_2. Consequently, the same leads to increased production of H_2O_2. This was established by quantitative measurement of superoxide radical in the catalyst dispersion.

We observed 3 distinct self-activation regions as shown in **Figure 2e** in manuscript and the **Figure below**: at first, a slow increase in activity for up to 5-6 days, then a rapid increase until 15-16 days, followed by a slow increase again before becoming constant in activity. It is believed that at the beginning, the ion leaching from the catalyst surface and defect formation is slow since the surface is highly crystalline, leading to a low rate of activation. However, after 6-5 days, as the surface becomes less crystalline and rich in defects, further vacancy formation becomes easier, more and more vacancy clusters form, and then the activation rate increases. Once the nanoplate surface is abundant in defects, further leaching cannot increase activation at a faster rate because leaching from only the crystalline region is primarily responsible for activation (the other regions are already more active now), resulting in a slow pace of activation. Ultimately, the surface becomes saturated with defects and the activity becomes consistent. Besides molecular oxygen activation, we found other factors, too, as the origin of the increase in catalytic activity, i.e. improved (i) light absorption and (ii) charge transport, as a consequence of defect formation. Therefore, the pace of increase in H_2O_2 production can be correlated, but in a complex manner, to the rate of formation of the surface vacancy associates.

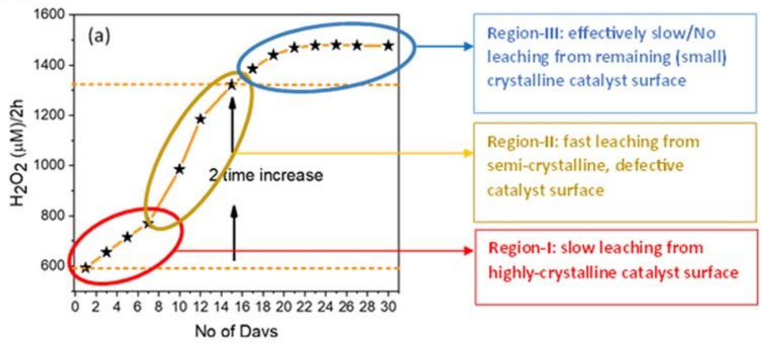

Figure showing non-linear performance enhancement of SBTOC-F during continuous use for 30 days of H_2O_2 production

Table S1. A comparison table showing photocatalytic H_2O_2 evolution activity of $Sr_2Bi_3Ta_2O_{11}Cl$ nanoplate as compared to other reported catalysts.[9–21]

Catalyst	Light Sources	Scavenger	H2O2 (mmol/h/g)	Atmosphere	References
SBTOC-F (Activated)	400 W Xe Lamp	Ethanol (10 vol%)	6.5	Air	**This Work**
SBTOC-F	400 W Xe Lamp		2.5	Air	
BiOCl	500 W Xe Lamp	HCOOH	0.68	Air	09
Au/Bi_2O_3-TiO_2	300 W Xe Lamp	Ethanol (4wt%)	0.9	Air	10
$Au_{0.1}Ag_{0.4}/TiO_2$	450 W high pressure Hg Lamp	Ethanol (4wt%)	0.3	O_2	11
CFT	300 W Xe Lamp		1.67	O_2	12
ZrS_3 Nanobelts	300 W Xe Lamp	Benzyl alcohol (1 mmol)	1.5	O_2	13
NH_2-MIL-125@ZnS	300 W Xe Lamp	Benzyl alcohol (1 ml) and acetonitrile (4 ml)	0.4	O_2	14
Sn-GQD/TiO_2	500 W Xe Lamp	2-propanol	1.7	O_2	15
$Bi_4O_5Br_2$/g-C_3N_4	300 W Xe Lamp		0.124	O_2	16
MMO@C_3N_4	300 W Xe Lamp		0.043	O_2	17
rGO/TiO_2	300 W Xe Lamp	propanol	0.533	O_2	18
PM-CDs-30	>400 W Xe Lamp		1.770	Air	19
SN-GQD/TiO_2	500 W Xe Lamp	2-propanol	0.92	O_2	20
CNP	300 W Xe Lamp	Ethanol	3.2	O_2	21

4. Supplemental References:

1 H. Luitel, A. Sarkar, M. Chakrabarti, S. Chattopadhyay, K. Asokan and D. Sanyal, *Nucl Instrum Methods Phys Res B*, 2016, **379**, 215–218.

2 H. Luitel, D. Sanyal, N. Gogurla and A. Sarkar, *J Mater Sci*, 2017, **52**, 7615–7623.

3 P. Sampathkumaran, S. Seetharamu, C. Ranganathaiah, J. M. Raj, P. K. Pujari, P. Maheshwari, D. Dutta and S. Kishore, *J Surf Eng Mater Adv Technol*, 2011, **01**, 136–143.

4 Y. Zhang, C. Zhou, S. Xu, H. Abdelsalam, Z. Mu, W. Chen, Z. Chen, X. Cheng, D. Khalafallah and Q. Zhang, *J Mater Chem A Mater*, 2023, **12**, 354–363.

5 X. Wang, K. Huang, L. Yuan, S. Li, W. Ma, Z. Liu and S. Feng, *ACS Appl Mater Interfaces*, 2018, **10**, 28219–28231.

6 L. Li, Q. Han, L. Tang, Y. Zhang, P. Li, Y. Zhou and Z. Zou, *Nanoscale*, 2018, **10**, 1905–1911.

7 S. Liu, P. E. R. Blanchard, M. Avdeev, B. J. Kennedy and C. D. Ling, *J Solid State Chem*, 2013, **205**, 165–170.

8 S. Mondal, S. R. Das, L. Sahoo, S. Dutta and U. K. Gautam, *J Am Chem Soc*, 2022, **144**, 2580–2589.

9 Y. Su, L. Zhang, W. Wang and D. Shao, *ACS Sustain Chem Eng*, 2018, **6**, 8704–8710.

10 L. Feng, B. Li, Y. Xiao, L. Li, Y. Zhang, Q. Zhao, G. Zuo, X. Meng and V. A. L. Roy, *Catal Commun*, 2021, **155**, 106315.

11 D. Tsukamoto, A. Shiro, Y. Shiraishi, Y. Sugano, S. Ichikawa, S. Tanaka and T. Hirai, *ACS Catal*, 2012, **2**, 599–603.

12 K. H. Kim, S. J. Kim, W. H. Choi, H. Lee, B. C. Moon, G. H. Kim, J. W. Choi, D. G. Park, J. H. Choi, H. Kim and J. K. Kang, *Adv Energy Mater*, 2022, **12**, 2104052.

13 Z. Tian, C. Han, Y. Zhao, W. Dai, X. Lian, Y. Wang, Y. Zheng, Y. Shi, X. Pan, Z. Huang, H. Li and W. Chen, *Nat Commun*, 2021,**12**, 2039.

14 C. Liu, T. Bao, L. Yuan, C. Zhang, J. Wang, J. Wan and C. Yu, *Adv Funct Mater*, 2022, **32**, 2111404.

15 L. Zheng, H. Su, J. Zhang, L. S. Walekar, H. Vafaei Molamahmood, B. Zhou, M. Long and Y. H. Hu, *Appl Catal B*, 2018, **239**, 475–484.

16 X. Zhao, Y. You, S. Huang, Y. Wu, Y. Ma, G. Zhang and Z. Zhang, *Appl Catal B*, 2020, **278**, 119251.

17 S. Li, J. A. Willoughby and O. J. Rojas, *ChemSusChem*, 2016, **9**, 2460–2469.

18 G. H. Moon, W. Kim, A. D. Bokare, N. E. Sung and W. Choi, *Energy Environ Sci*, 2014, **7**, 4023–4028.

19 Q. Wu, J. Cao, X. Wang, Y. Liu, Y. Zhao, H. Wang, Y. Liu, H. Huang, F. Liao, M. Shao and Z. Kang, *Nat Commun*, 2021, **12**, 483.

20 H. il Kim, Y. Choi, S. Hu, W. Choi and J. H. Kim, *Appl Catal B*, 2018, **229**, 121–129.

21 H. Yang, C. Li, T. Liu, T. Fellowes, S. Y. Chong, L. Catalano, M. Bahri, W. Zhang, Y. Xu, L. Liu, W. Zhao, A. M. Gardner, R. Clowes, N. D. Browning, X. Li, A. J. Cowan and A. I. Cooper, *Nat Nanotechnol*, 2023, **18**, 307–315.
